# Psoriatic Arthritis and Sarcopenia: A Cross-Sectional Ultrasonographic Study

**DOI:** 10.5152/ArchRheumatol.2025.11084

**Published:** 2025-09-01

**Authors:** Gonca Canan Doğan Tosun, Tuba Güler, Fatma Gül Yurdakul, Hatice Bodur, Mehtap Balaban

**Affiliations:** 1 Ankara City Hospital, Physical Medicine and Rehabilitation Hospital, Ankara, Türkiye; 2 Yıldırım Beyazıt University Faculty of Medicine, Ankara City Hospital Physical Medicine and Rehabilitation Hospital, Ankara, Türkiye

**Keywords:** Psoriatic arthritis, rheumatic disease, sarcopenia, ultrasonography

## Abstract

**Background/Aims:**

This study aims to identify sarcopenia and its associated factors in patients with psoriatic arthritis (PsA) and to assess the diagnostic utility of ultrasonography (USG) for identifying sarcopenia.

**Materials and Methods:**

The study included 54 PsA patients (21 males and 33 females; mean age: 46.5 ± 10.93; range, 18-65) and 55 age-, gender- and body mass index (BMI)–matched healthy controls (19 males and 36 females; mean age: 48 ± 11.30; range, 18-65). Demographic data, anthropometric measurements, functional assessments, handgrip strength, and 4-meter gait speed were evaluated. Disease activity was evaluated using the psoriatic arthritis impact of disease 12-item questionnaire (PSAID12), disease activity score 28 (DAS28), bath ankylosing spondylitis disease activity index (BASDAI), disease activity in psoriatic arthritis (DAPSA), and skin lesions with the psoriasis area and severity index (PASI). The thickness of bilateral rectus femoris, vastus intermedius, and quadriceps muscle were measured using USG. Whole-body muscle mass was analyzed via dual-energy x-ray absorptiometry.

**Results:**

Sarcopenia was diagnosed in 22 PsA patients (40.7%) and 12 healthy controls (21.8%). An association between sarcopenia, BMI, and disease duration was identified (*P* < .05). It was not associated with PSAID12, DAS28, BASDAI, DAPSA, PASI, age, gender, comorbidities, smoking, alcohol consumption, erythrocyte sedimentation rate, C-reactive protein, vitamin D levels, and history of falls. The USG measurements revealed that PsA patients with sarcopenia had lower thickness of rectus femoris, vastus intermedius, and quadriceps muscles (*P *< .05). Receiver-operating characteristic analysis was performed to determine the diagnostic cut-off values, which were as follows: right/left rectus femoris, 1.11 cm; right vastus intermedius, 1.17 cm; left vastus intermedius, 1.19 cm; right quadriceps, 2.31 cm; left quadriceps, 2.32 cm.

**Conclusion:**

The presence of sarcopenia was higher in PsA patients compared to healthy controls. The USG may be a practical and acceptable method for assessing muscle mass and diagnosing sarcopenia in patients with PsA.

**Main Points**

- The presence of sarcopenia was higher in PsA patients compared to healthy controls.
- Sarcopenia showed a statistically significant association with both BMI and disease duration in PsA patients.
- Ultrasonographic measurements demonstrated reduced rectus femoris, vastus intermedius, and quadriceps muscle thickness in PsA patients with sarcopenia.
- USG may be a practical and acceptable method for assessing muscle mass and diagnosing sarcopenia in patients with PsA.

## Introduction

Psoriatic arthritis (PsA) is a chronic inflammatory disorder that presents with a wide range of clinical features, including both peripheral and axial joint involvement, enthesitis, dactylitis, and characteristic skin and nail changes, all of which may contribute to considerable disability.^1^

Sarcopenia is a chronic neuromuscular disorder associated with aging. It is defined by a progressive decline in skeletal muscle mass (SMM) and function, accompanied by impaired physical performance. This condition is marked by reduced mobility and is linked to an increased risk of adverse clinical outcomes, including higher rates of morbidity and all-cause mortality.^2^ Although sarcopenia is commonly associated with aging; immobilization, malnutrition, and chronic diseases can also contribute to its development.^3^ Muscle loss due to chronic diseases is more progressive compared to age-related muscle loss.^4^ Sarcopenia is diagnosed based on the evaluation of muscle mass. Methods such as ultrasonography (USG), bioelectrical impedance analysis, dual-energy X-ray absorptiometry (DEXA), computed tomography, and magnetic resonance imaging (MRI) are employed to assess muscle mass. Once sarcopenia is confirmed, physical performance assessments such as gait speed, the short physical performance battery, and the timed up and go test are used to identify severe sarcopenia.^5^

Muscle USG has emerged as a new method for the diagnosis of sarcopenia. Regardless of the diagnostic criteria, measurement of muscle mass remains a cornerstone in the diagnosis of sarcopenia. Owing to its safety, non-invasiveness, low cost, and real-time capability, USG has gained increasing prominence as a tool for muscle mass measurement. The USG is considered a highly reproducible, accurate, and reliable method for measuring muscle mass.^6^ In a meta-analysis conducted by Fu et al,^7^ lower extremity muscle thickness was evaluated as a common parameter for diagnosing sarcopenia using USG.

Although sarcopenia has been investigated in various chronic inflammatory conditions, studies specifically involving PsA patients remain scarce. Most of the current USG literature pertains to individuals with rheumatoid arthritis (RA), with limited evidence available on its application for assessing sarcopenia in PsA. This study is therefore of particular significance, as it represents the first to employ USG in evaluating sarcopenia in patients with PsA.

## Methods

### Study Design

This cross-sectional case-control study was performed between September 2022 and September 2023 at Ankara City Hospital Physical Therapy and Rehabilitation. The study was conducted following approval from the Ethics Committee for Clinical Research of the University of University of Health Sciences (Approval date: September 14, 2022; Approval number: E2-22-2238) and in compliance with the principles outlined in the Declaration of Helsinki. Each individual voluntarily signed a written consent form before being enrolled in the study.

### Participants

A total of 109 participants took part in the study. Among them, 54 patients (21 males, 33 females; mean age: 46.5 ± 10.93 years), aged between 18 and 65 years, had been diagnosed with PsA in accordance with the CASPAR criteria at least 1 year earlier and had experienced no modifications to their therapeutic regimen within the preceding 3 months. The control group consisted of 55 age, gender, and body mass index (BMI)-matched healthy individuals. The exclusion criteria included the following: (1) individuals with neurological diseases; (2) hip dislocation; (3) upper and lower extremity deformities; (4) joint arthroplasty in the upper and lower extremities; (5) lumbar stabilization; (6) cognitive impairments preventing participation; (7) participants exceeding 100 kg in body weight were excluded from the study due to the device’s maximum measurable weight limit; and (8) individuals who did not consent to participate.

### Clinical Assessment

Demographic data of the patients (age, gender, education level, occupation, and marital status), medication use, history of rheumatological disease, time of PsA diagnosis, disease duration, and presence of chronic disease were recorded. Additionally, smoking status, weight, height, BMI, erythrocyte sedimentation rate (ESR), C-reactive protein (CRP), vitamin D levels, and history of falls were documented.

### Anthropometric Assessment

Anthropometric measurements of PsA patients and healthy controls (mid-upper arm circumference of both arms and the largest circumference of both calves) were performed.

Height and weight were documented for all participants in both groups, and BMI values were computed accordingly.

### Muscle Strength Assessment

Handgrip strength was assessed with a Jamar hydraulic hand dynamometer (FEI®, model 5030J1, USA) with the participant positioned in shoulder adduction, elbow at 90 degrees of flexion, and the forearm and wrist in a neutral, supported position.^8^ A study conducted by Gulistan Bahat et al^9^ in 2016 aimed to define reference cut-off values for the Turkish population to improve the general applicability of EWGSOP criteria. The study found handgrip strength cut-off values for sarcopenia to be 32 kg for males and 22 kg for females. In the present study, handgrip strength below 32 kg for males and 22 kg for females was considered reduced handgrip strength.

### Physical Performance Assessment

A 4-meter gait speed test (including 1 meter acceleration, 4-meter walking area, and 1 meter deceleration area) was used to assess physical performance in both groups. A walking speed of <0.8 m/s was interpreted as impaired physical function.^10^

### Muscle Mass Assessment

To assess muscle mass in both groups, quadriceps muscle thickness of the dominant and non-dominant limbs was measured using a Logiq 9 ultrasound device (GE, USA) with a high-frequency 7-12 MHz linear probe. Muscle thickness was measured bilaterally in the distal 1/3 of the distance between the superior anterior iliac spine and the superior pole of the patella in a seated position. Axial images were recorded after ensuring no compression on subcutaneous fat and muscle tissue.

Whole-body muscle mass was assessed using DEXA measurement results obtained from previously conducted scans (Lunar iDEXA; GE Healthcare, 3030 Ohmeda Drive, Madison, WI 53718). Appendicular skeletal muscle mass (ASM) was first calculated, and the SMM index (SMMI) was then derived using the ASM/height^2^ formula. An SMMI value below 7.4 for females and 9.2 for males was considered significant.^9^

### Assessment of Activities of Daily Living, Balance-Gait, and Psychosocial Characteristics

Sarcopenia quality of life questionnaire (SarQoL) consisting of 55 items in 22 questions,^11^ Tinetti balance and gait test,^12^ and the hospital anxiety and depression scale^13^ were administered to both groups.

### Assessment of Disease Activity

Disease activity in patients with PsA was evaluated using the psoriatic arthritis impact of disease 12-item questionnaire,^14^ disease activity score 28 (DAS28),^15^ bath ankylosing spondylitis disease activity index (BASDAI),^16^ and disease activity in psoriatic arthritis (DAPSA)^17^ as well as skin lesions with the psoriasis area and severity index (PASI).^18^

### Statistical Analysis

Sample size estimation and power calculation were executed using G*Power software version 3.1.9.4 (Heinrich Heine University, Düsseldorf, Germany). The analysis indicated that at least 53 individuals per group (total n = 106) were necessary to achieve a statistical power of 0.80. IBM SPSS Statistics for Windows version 23.0 (IBM SPSS Corp.; Armonk, NY, USA) was used for data analysis. Normal distribution of numerical data was examined using the Kolmogorov–Smirnov test. General descriptive statistics were presented as mean, median, standard deviation, minimum, and maximum values for continuous variables, and as count and percentage (%) for categorical variables. Categorical variables were compared using the Chi-square test or Fisher’s exact test, and continuous variables were analyzed using the Student’s *t*-test or Mann–Whitney *U* test based on data distribution. The relationship between ultrasonographic muscle thickness and SMMI was analyzed using Pearson correlation analysis. The receiver-operating characteristic (ROC) curve analysis was performed to calculate cut-off values for sarcopenia-related thickness of the right and left rectus femoris, vastus intermedius, and quadriceps muscles. All analyses were conducted with a 95% CI, and statistical significance was defined as *P* < .05.

## Results

A total of 109 participants were included in this study, consisting of 54 PsA patients (mean age: 46.5 ± 10.93 years) and 55 healthy controls (mean age: 48 ± 11.30 years). Table 1 summarizes the demographic characteristics of the participants. There were no statistically significant differences between the groups regarding age, gender, occupation, or education.

The average disease duration of PsA patients was 10.24 ± 10.28 years. Axial involvement was present in 24 patients (44%), while oligoarticular involvement was observed in 30 patients (56%). A total of 29 patients were found to use conventional synthetic disease-modifying antirheumatic drugs (csDMARDs), 11 patients were using biological DMARDs, 3 patients were taking a combination of csDMARDs and bDMARDs, 7 patients were taking only NSAIDs, and 1 patient were taking only steroids. Based on DAPSA scores, 3.7% of patients (n = 2) were classified as being in remission, while 24.2% (n = 13) exhibited low, 33.3% (n = 18) moderate, and 38.8% (n = 21) high levels of disease activity. According to BASDAI, 37 (68.5%) patients were classified as having active disease.

Functional assessments were conducted in both the PsA group and healthy controls. The PsA patients exhibited lower dominant and non-dominant handgrip strength, reduced 4-meter gait speed, decreased SarQoL scores, and smaller right calf circumference in comparison to the healthy control group (*P* < .05).

While the difference in Tinetti balance test scores between the 2 groups was not statistically significant, the Tinetti gait test scores were lower in the PsA patient group (*P* < .05). No statistically significant differences were observed between the 2 groups in terms of the hospital anxiety and depression scale results (Table 2).

Sarcopenia was diagnosed in 22 PsA patients (40.7%) and 12 healthy controls (21.8%) based on the SMMI cut-off value^9^ (*P* < .05). Associations between sarcopenia and clinical variables were analyzed. There was no meaningful association identified between sarcopenia and variables such as age, gender, comorbidities, smoking, alcohol consumption, disease activity, ESR, CRP, vitamin D levels, or history of falls. However, sarcopenia was found to be associated with BMI and disease duration (*P *= .025, *P *= .017 , respectively) (Table 3).

The quadriceps muscle thickness measurements were lower in sarcopenic PsA patients while there were no statistically significant differences in subcutaneous tissue between sarcopenic and non-sarcopenic individuals (Table 4).

The ultrasonographic muscle thickness of the right and left vastus intermedius and quadriceps showed a positive correlation with SMMI (*r* = 0.444, *P *< .001; *r *= 0.463, *P *< .001; *r *= 0.364, *P *= .007; *r *= 0.369, *P *= .006, respectively). The correlation between SMMI and both disease duration and BMI was also examined. For BMI and SMMI, *r* = 0.675, *P* < .001; for disease duration and SMMI, *r* = 0.318, *P* = .318.

**Figure 1.**
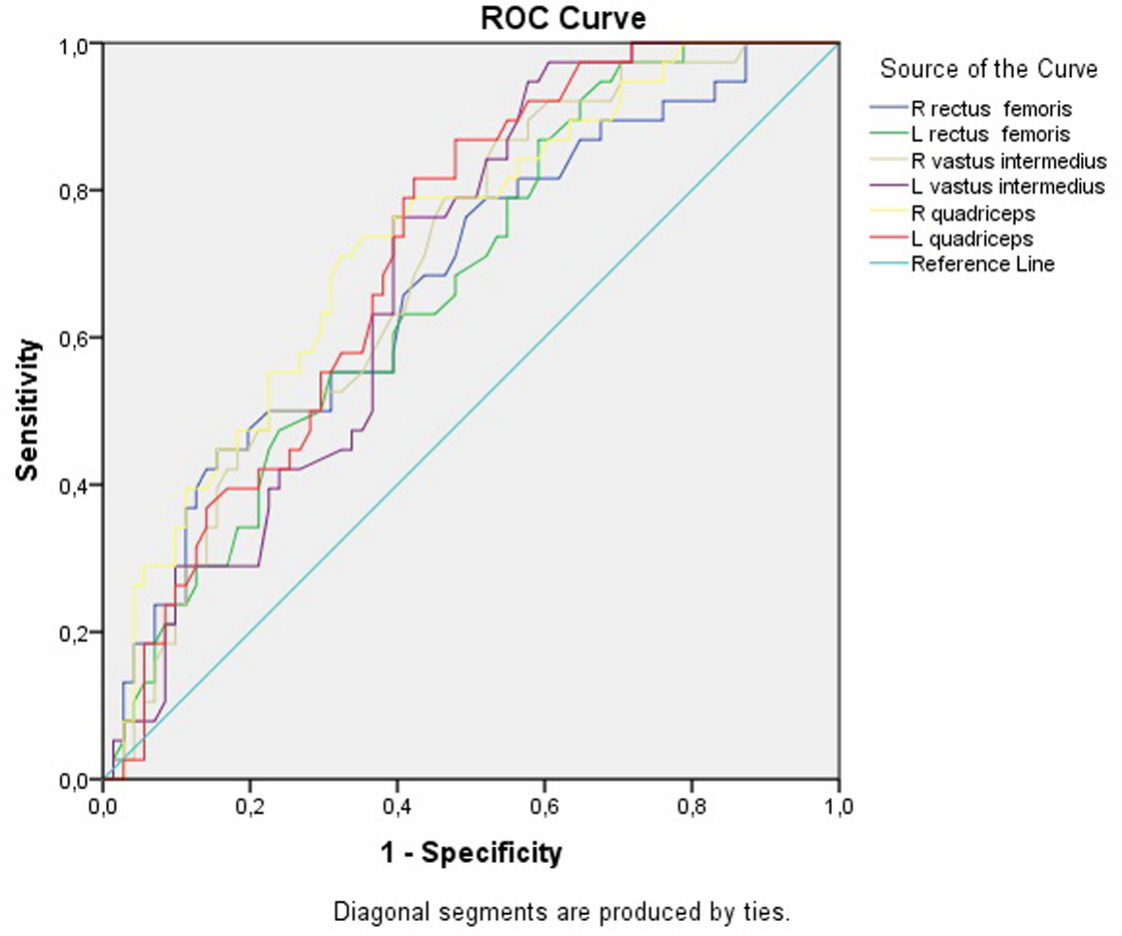
Diagonal segments are produced by ties.

The ROC analysis was employed to assess the diagnostic performance of muscle thickness measurements in identifying sarcopenia. Results of ROC analysis for ultrasonographic muscle thickness are presented in Table 5. The cut-off value for the right and left rectus femoris muscle thickness was determined to be 1.11 cm (sensitivity was 57%, and specificity was 60%). The cut-off value for the right vastus intermedius muscle thickness was 1.17 cm, (sensitivity of 61% and specificity of 62%). For the left vastus intermedius muscle thickness, the cut-off value was 1.19 cm, with a sensitivity of 63% and specificity of 62%. The cut-off value for the right quadriceps muscle thickness was determined to be 2.31 cm, with a sensitivity of 68% and specificity of 69%. For the left quadriceps muscle thickness, the cut-off value was 2.32 cm, with a sensitivity of 68% and specificity of 62%. The results of the ROC analysis are shown in Figure 1.

## Discussion

In this study investigating sarcopenia in patients with PsA, the presence of sarcopenia was found to be 40.7% among PsA patients compared to 21.8% in healthy controls. A significant association was observed between sarcopenia and both BMI and disease duration. However, no statistically significant correlations were observed with age, gender, disease activity, comorbidities, history of falls, smoking status, alcohol consumption, or vitamin D levels. Diagnostic cut-off values for the thickness of the rectus femoris, vastus intermedius, and quadriceps muscles were established through ROC curve analysis.

The number of studies exploring sarcopenia in PsA is limited. Existing research has reported the prevalence of sarcopenia to be between 9.1% and 49% in individuals with PsA.^19-21^ In studies examining inflammatory diseases and sarcopenia, the prevalence has been found to be between 7.8% and 39.8% in patients with RA,^19,20,22-24^ and between 1.7% and 61.7% in those with spondyloarthritis (SpA).^19,25,26^ The differences in reported sarcopenia rates across studies can be attributed to variations in the criteria, evaluation methods, and sample sizes used in each study.

In this study, sarcopenia in PsA patients was found to be associated with disease duration. However, the literature presents conflicting evidence on this relationship. For instance, Barone et al^20^ and the SASPAR study^25^ reported no association between disease duration and sarcopenia in PsA and SpA patients, respectively. Conversely, a meta-analysis^27^ and comprehensive review^28^ demonstrated a link between disease duration and sarcopenia in RA patients. Similarly, Torii et al^24^ identified disease duration as a factor associated with sarcopenia in RA. Aligning with these findings, the present study confirms the association, likely reflecting prolonged exposure to systemic chronic inflammation.

The present study identified a significant association between low BMI and sarcopenia, corroborating earlier research reporting comparable findings.^22,23^ Taken together, the evidence emphasizes the crucial contribution of undernutrition and malnutrition to the onset of sarcopenia.

As part of the current study, no relevant link was identified between sarcopenia and indicators of disease activity, including PSAID-12, DAS28, DAPSA, BASDAI, and PASI. There is currently no other study in the literature that has investigated the relationship between disease activity and sarcopenia in individuals with PsA. However, findings on this relationship remain inconsistent in studies involving RA and SpA. In the SASPAR study,^25^ BASDAI was found to be associated with sarcopenia. Mochizuki et al^22^ reported no significant association between DAS28-ESR and sarcopenia in patients with RA. In the meta-analysis^27^ DAS28 values were emphasized to be associated with sarcopenia in RA patients. In this study, the absence of an association between sarcopenia and disease activity may be attributed to the cross-sectional evaluation of disease activity.

Research interest in the link between sarcopenia and rheumatic diseases has grown substantially in recent years. Chronic inflammatory activity is believed to be a key mechanism, with pro-inflammatory cytokines contributing to impaired skeletal muscle function, disrupted protein synthesis, and loss of muscle mass.^29^ Tumor necrosis factor-alpha, a key cytokine involved in PsA, drives inflammation in the synovium, entheses, and bone, and is also implicated in muscle aging and the development of sarcopenia.^29^ Likewise, prolonged elevation of interleukin-6 has been shown to promote muscle wasting, trigger catabolic pathways, and reduce muscle strength.^30^ It has been proposed that biological DMARD therapy may exert a protective role against the onset of sarcopenia.^31^ Torii et al^24^ reported a negative correlation between bDMARDs treatment and sarcopenia. However, Barone et al^20^ argued that biologic therapy does not have a protective effect against sarcopenia. In the meta-analysis by Hein et al,^32^ which evaluated the impact of DMARDs on muscle mass in individuals with RA, no significant influence of DMARD use on muscle mass was observed. In this study, neither anti-TNF agents nor other biologic therapies demonstrated a statistically significant impact on sarcopenia. Additional research is needed to better elucidate this association.

In the present study, no association was found between sarcopenia and gender. While some studies have reported higher rates of sarcopenia in males^20,25^ others have identified a greater prevalence in females.^26^ However, limited data restricts a definitive conclusion on the gender-sarcopenia relationship. Similarly, no link was observed between vitamin D levels and sarcopenia in our study. Minamino et al,^33^ in a study of female patients with RA, found that low vitamin D levels were associated with severe sarcopenia, reduced muscle mass, weaker grip strength, and slower gait speed. In contrast, most participants in the current study had either completed or were receiving vitamin D supplementation, which may explain the absence of a significant association.

In the present study, USG was employed to assess the bilateral muscle thickness of the rectus femoris, vastus intermedius, as well as the total quadriceps thickness. The analysis demonstrated that individuals diagnosed with sarcopenia exhibited a markedly reduced thickness across all 3 muscle groups compared to healthy controls. The area under the ROC curve and the corresponding cut-off values for rectus femoris thickness were identical bilaterally, measured at 1.11 cm for both the right and left legs. For the vastus intermedius, the cut-off thresholds were 1.17 cm on the right side and 1.19 cm on the left. Meanwhile, the quadriceps muscle exhibited cut-off values of 2.31 cm on the right and 2.32 cm on the left. These findings align with existing literature. In a study conducted by Tada et al,^34^ ultrasound-based assessment of thigh muscle and adipose tissue thickness in patients with RA demonstrated a significant reduction in quadriceps thickness among sarcopenic individuals. Similarly, Salaffi et al^35^ reviewed the utility of ultrasound and MRI in detecting sarcopenia within immune-mediated rheumatic diseases, reporting that RA patients exhibited decreased vastus lateralis fascicle thickness and length compared to healthy controls. Furthermore, Dos Santos et al^36^ employed ultrasound-based assessment of quadriceps thickness in RA patients, revealing not only reduced muscle thickness but also concomitant declines in muscle strength.

This study has several limitations, including its cross-sectional design and relatively small sample size. Future longitudinal studies with larger cohorts are warranted to further elucidate the relationship between PsA and sarcopenia.

A key strength of this study is the novel application of USG to evaluate sarcopenia in patients with PsA, which is expected to contribute meaningfully to the current literature.

To conclude, the findings of this study highlight that sarcopenia is more prevalent among individuals with PsA. In patients with PsA, sarcopenia was significantly associated with BMI and disease duration. Ultrasonographic assessments revealed that the sarcopenic group exhibited notably reduced thickness of the rectus femoris, vastus intermedius, and quadriceps muscles. The ROC analyses identified specific cut-off values for these muscle groups, supporting their diagnostic relevance. Given its accessibility, safety, and ease of application, USG may be considered a practical and supportive modality for the assessment of sarcopenia in clinical settings.

## Figures and Tables

**Table 1.** Demographic Characteristics of Psoriatic Arthritis Patients and Control Group

	PsA	Control	*P*
Age, years, mean ± SD	46.5 ± 10.9	48 ± 11.3	.956
Gender, n (%) Female Male	33 (61.1) 21 (38.9)	36 (65.5) 19 (34.5)	.638
Body mass index, kg/m^2^, mean ± SD	29.0 ± 5.7	27.3 ± 4.7	.094
Occupation, n (%) Office worker Worker Housewife Retired	22 (40.7) 7 (13) 22 (40.7) 3 (5.6)	29 (52.7) 8 (14.5) 15 (27.3) 3 (5.5)	.504
Education, n (%) Primary school Secondary school High school Associate degree License	17 (31.5) 5 (9.3) 13 (24.1) 3 (5.6) 16 (29.6)	16 (29.1) 3 (5.5) 16 (29.1) 2 (3.6) 18 (32.7)	.886
Dominant hand, n (%)	54 (100)	55 (100)	–
Smoking, n (%) Never Active Ex-smoker	29 (53.7) 23 (42.6) 2 (3.7)	39 (70.9) 15 (27.3) 1 (1.8)	.176
Cigarettes (pack/year) median (min-max)	0 (0-40)	0 (0-35)	.191
Alcohol consumption, n (%) Yes No	4 (7.4) 50 (92.6)	5 (9.1) 55 (90.9)	.573
Comorbidity, n (%) Yes No	33 (61.1) 21 (38.9)	22 (40) 33 (60)	**.028**
Comorbidity, n (%) No HT DM The history of CAD Hypothyroidism HT + DM Other	21 (36.6) 10 (17.4) 1 (1.9) 3 (5.2) 4 (7.4) 3 (5.6) 12 (25.9)	32 (58.2) 10 (18) 1 (1.8) 1 (1.8) 5 (9.1) 1 (1.8) 5 (9.1)	.195
Vitamin D, median (min-max), nmol/L	30 (8.5-92)	33 (6-98)	.132

Of the data, the normally distributed ones were given as ± SD and the non-normally distributed ones were given as median (min-max). *P *< .05 was considered significant.

BMI, body mass index; CAD, coronary artery disease; DM, diabetes mellitus; HT, hypertension; PsA, psoriatic arthritis.

**Table 2.** Functional Assessments of Patients with Psoriatic Arthritis and Control Group

	PsA	Control	*P*
Muscle strength (handgrip strength) Dominant hand median (min-max) Non-dominant hand median (min-max)	20 (2-70) 20 (2-70)	30 (10-50) 25 (10-48)	**.004** **<.001**
4-meter gait speed test median (min-max) (m/s)	1.1 (0.50-2.50)	1.4 (0.67-2.34)	**.004**
SARQoL test (mean ± SD)	60.1 ± 15.8	75.9 ± 13.9	**<.001**
Tinetti Balance test median (min-max) Gait test median (min-max)	26 (17-29) 9 (6-9)	26 (23-26) 9 (9-9)	.147 **.041**
Depression-anxiety scale median (min-max) Depression scale median (min-max) Anxiety scale median (min-max)	5 (0-20) 7.5 (1-19)	5 (0-16) 5 (0-17)	.342 .078

From the data, the normally distributed ones were given as ± SD and the normally distributed ones were given as median (min-max). *P *< .05 was considered significant.

PsA, psoriatic arthritis; SARQoL, sarcopenia quality of life questionnaire.

**Table 3.** Variables Associated with Sarcopenia in Sarcopenic Patients with Psoriatic Arthritis

	Sarcopenia	Non-Sarcopenia	*P*
Age (mean ± SD)	44.2 ± 11.5	46.6 ± 10.5	.433
Gender n (%) Female Male	22 (40.7) 15 (68.2) 7 (31.8)	32 (59.3) 18 (56.3) 14 (43.8)	.377
BMI (mean ± SD)	26.4 ± 3.5	30.7 ± 6.3	**.025**
Comorbidity Yes No	15 (68.2) 7 (31.8)	18 (56.3) 14 (43.8)	.377
Smoking n (%) Never Active Ex-smoker	12 (45.5) 10 (54.5) 0 (0)	13 (40.6) 17 (53.1) 0 (0)	.484
Cigarettes (packs/year) med (min-max)	0.0 (0-40)	0.0 (0-30)	.822
Alcohol consumption n (%) Yes No	1 (4.5) 21 (95.5)	3 (9.4) 29 (90.6)	.506
Disease duration median (min-max) (years)	10.5 (1-35)	4 (1-50)	**.017**
Medication n (%) bDMARDs-TNF-α inhibitors non-TNF-α bDMARDs csDMARDs/NSAID/corticosteroid	5 (22.7) – 17 (77.3)	8 (25.0) 3 (9.4) 21 (65.6)	.310
DAS-28 (mean ± SD)	3.6 ± 1.1	3.2 ± 1.2	.267
DAPSA (mean ± SD)	31.3 ± 21.9	25.2 ± 20.1	.190
BASDAI (mean ± SD)	5.7 ± 2.2	4.8 ± 2.6	.218
PSAID-12 (mean ± SD)	4.7 ± 2.4	4.3 ± 2.1	.567
PASI median (min-max)	0 (0-32.4)	1.5 (0-21.9)	.085
ESR median (min-max) (mm/h)	11.5 (1-45)	7.5 (3-31)	.132
CRP median (min-max) (mg/dL)	4 (0-61)	3 (0-32)	.101
Vitamin D median (min-max) (nmol/L)	39 (14.2-82)	29 (8.5-92)	.452
History of falls n (%) Yes No	4 (18.2) 18 (81.8)	5 (15.6) 27 (84.4)	.804
Right calf circumference (mean ± SD)	35.8 ± 3.1	37.9 ± 3.8	**.049**

From the data, the normally distributed ones were given as ± SD and the normally distributed ones were given as median (min-max). *P *< .05 was considered significant.

BASDAI, bath ankylosing spondylitis disease activity index; bDMARDs, biologic disease-modifying antirheumatic drugs; BMI, body mass index (kg/m^2^); CRP, C-reactive protein (mg/dL); csDMARDs, conventional synthetic disease-modifying antirheumatic drugs; DAS-28, disease activity score; DAPSA, disease activity in psoriatic arthritis; ESR, erythrocyte sedimentation rate (mm/h); NSAID, non-steroidal anti-inflammatory drug; PASI, psoriasis area severity index; PSAID-12, psoriatic arthritis impact of disease 12-item questionnaire; TNF, tumor necrosis factor.

**Table 4.** USG Measurements of Sarcopenic and Non-Sarcopenic Psoriatic Arthritis Patients

USG Thickness Measurements (cm)	Sarcopenia	Non-Sarcopenia	*P*
Right subcutaneous tissue (mean ± SD)	0.97 ± 0.35	1.00 ± 0.54	.826
Left subcutaneous tissue (mean ± SD)	0.99 ± 0.98	1.00 ± 0.52	.666
Right rectus femoris (mean ± SD)	1.06 ± 0.28	1.27 ± 0.37	**.040**
Left rectus femoris (mean ± SD)	1.01 ± 0.31	1.29 ± 0.39	**.006**
Right vastus intermedius (mean ± SD)	1.11 ± 0.31	1.31 ± 0.37	**.048**
Left vastus intermedius (mean ± SD)	1.15 ± 0.32	1.35 ± 0.34	**.028**
Right quadriceps (mean ± SD)	2.17 ± 0.52	2.59 ± 0.63	**.016**
Left quadriceps (mean ± SD)	2.17 ± 0.59	2.64 ± 0.60	.**004**

From the data, the normally distributed ones were given as ± SD and the normally distributed ones were given as median (min-max). *P *< .05 was considered significant

USG, ultrasonography.

Values with *P* < 0.05 are indicated in bold

**Table 5.** ROC Analysis Result of Ultrasonographic Muscle Thickness

USG thickness measurements (cm)	Area	Standard Error	**P**	CI (95%)	
				Lower Bound	Upper Bound
Right rectus femoris	0.680	0.053	.001	0.575	0.784
Left rectus femoris	0.672	0.052	.001	0.570	0.773
Right vastus intermedius	0.697	0.050	<.001	0.598	0.795
Left vastus intermedius	0.691	0.050	<.001	0.594	0.788
Right quadriceps	0.731	0.049	<.001	0.634	0.827
Left quadriceps	0.716	0.048	<.001	0.622	0.811

*P *< .05 was considered significant

USG, ultrasonography.

## Data Availability

The data that support the findings of this study are available on request from the corresponding author.
